# Synergistic interaction and activation of the opioid receptor-NO–cGMP–K^+^ channel pathway on peripheral antinociception induced by the *α*-Bisabolol-diclofenac combination

**DOI:** 10.3389/fphar.2023.1158236

**Published:** 2023-04-13

**Authors:** Mario I. Ortiz

**Affiliations:** Área Académica de Medicina del Instituto de Ciencias de la Salud, Universidad Autónoma del Estado de Hidalgo, Pachuca, Hidalgo, Mexico

**Keywords:** *α*-Bisabolol, diclofenac, synergism, nitric oxide, cGMP, opioid receptor, metformin, K^+^ channels

## Abstract

**Introduction:** The local peripheral combination of analgesic drugs with herbal derivatives may have beneficial effects. Information on the action mechanism of these interactions between drugs is scarce. Therefore, the main of the present study was to determine the pharmacological interaction and action mechanism of the combination α-Bisabolol and diclofenac.

**Methods:** Rats were injected in the dorsal surface of the right hind paw with 1% formalin. Rats received subcutaneous injections in the dorsal surface of paw of vehicles or increasing doses of α-Bisabolol, diclofenac or their combination before formalin injection into the paw. Antinociception of the α-Bisabolol + diclofenac combination was evaluated with and without the local treatment of naloxone, metformin, NG-nitro-L-arginine methyl ester (L-NAME), 1H- (1,2,4)-oxadiazolo (4,2-a) quinoxalin-1-one (ODQ), glibenclamide, glipizide, 4-aminopyridine, tetraethylammonium, apamin, or charybdotoxin.

**Results:** α-Bisabolol, diclofenac or α-Bisabolol-diclofenac combinations produced significant antinociception in the rat (*p* < 0.05). The experimental effective dose (ED) value of 109.2 µg/paw was different significantly of the theoretical effective dose (ED) of 245.7 µg/paw (synergism). Blockers significantly reverted the antinociception produced by the synergistic combination of α-Bisabolol and diclofenac.

**Discussion:** Data showed a synergism of the α-Bisabolol-diclofenac combination and the activation of the opioid receptor-Nitric Oxide–cyclic GMP–K^+^ channels pathway and a biguanide-dependent mechanism in order to produce the potentiation of its peripheral antinociception in the formalin test.

## 1 Introduction

α-Bisabolol or levomenol belongs to the family of sesquiterpenes, which is isolated from the essential oil of *Vanillosmopsis arborea* Barker (Asteraceae), *Nectandra megapotamicav* (Spreng.) Mez. (*Lauraceae*), *Matricaria chamomilla* L (German chamomile), among other medicinal plants (Barreto et al., 2016; Eddin et al., 2022). *α*-Bisabolol has antimicrobial, antitumor, neuroprotective, utero-relaxant, cardioprotective, anti-irritant, anti-inflammatory, gastroprotective and antinociceptive activities (Rocha et al., 2011a; Rocha et al., 2011b; da Silva et al., 2010; Muñoz-Pérez et al., 2018; Ortiz et al., 2018; Ortiz et al., 2021; Eddin et al., 2022). Diverse action mechanisms have been determined for some of the biological effects of *α*-Bisabolol, such as regulation of the expression of the inducible nitric oxide synthase and cyclooxygenase-2 genes, activation of the nitric oxide (NO)-cyclic guanosine monophosphate (cGMP)-K^+^ channel pathway, inhibition of nuclear factor kappa-light-chain-enhancer of activated B cells activation, interleukin-10 production, reduction of tumor necrosis factor alpha and interleukin-1β, among others (Rocha et al., 2011a; Rocha et al., 2011b; da Silva et al., 2010; Muñoz-Pérez et al., 2018; Ortiz et al., 2021; Eddin et al., 2022). *α*-Bisabolol is a moisturizing drug, which is used clinically as a skin conditioning and anti-inflammatory agent, which also protects against photodamage, decreases itching and the wound and ulcer surface area (Eddin et al., 2022). The cutaneous application of *α*-Bisabolol undergoes rapid penetration through the layers of the skin and has important systemic absorption (Hahn and Hölzl, 1987). The evidence about the action mechanisms at cutaneous or subcutaneous levels are scarce. A previous study found that the subcutaneous administration in the paw of the rat of *α*-Bisabolol produced peripheral antinociception through the activation of the NO-cGMP-K^+^ channels pathway, but not opioid receptors (Ortiz et al., 2021).

Diclofenac is a non-steroidal anti-inflammatory drug widely used for the treatment of inflammation and pain present in rheumatic conditions and those produced by tissue damage due to physical trauma or surgery (Wiffen and Xia, 2020). Unfortunately, diclofenac is associated with the presence of gastrointestinal (GI) and cardiovascular (CV) damages (Odom et al., 2014; van Walsem et al., 2015). A meta-analysis demonstrated that GI and CV lesions produced by diclofenac are dose-dependent; that is, the higher the dose administered, the greater the damage (Odom et al., 2014). Therefore, two strategies can be used to decrease the diclofenac-induced injuries, the first one recommends the use of low doses of diclofenac and the second one is its topical application. However, low doses of diclofenac may be less effective. Given this, a probable solution is the use of diclofenac at low doses in combination with another safe and effective analgesic and anti-inflammatory-drug. In this sense, several preclinical and clinical studies have demonstrated the effectiveness and security of diclofenac in combination with other analgesic drugs or drugs derived from plants, such as curcuminoids, *Matricaria chamomilla* or *Calendula officinalis* extracts, *α*-Bisabolol, methyl salicylate, pyrilamine or paracetamol (De Paz-Campos et al., 2014; Ortiz et al., 2016; Ortiz, 2017; Ortiz et al., 2017; Aweke et al., 2018; Ortiz et al., 2018; Jurca et al., 2020; Shep et al., 2020). Previous studies demonstrated systemic synergistic antinociceptive, anti-inflammatory and gastroprotective effects produced by the combination of diclofenac with the *Matricaria chamomilla* ethanolic extract or *α*-Bisabolol (Ortiz et al., 2017; Ortiz et al., 2018). There is no report that informs the action mechanism of the pharmacological interaction between diclofenac and the drugs used in combination. Therefore, the main of the present study was to determine the kind of peripheral pharmacological interaction and action mechanism of the combination *α*-Bisabolol and diclofenac in the 1% rat formalin test.

## 2 Materials and methods

### 2.1 Animals

Male Wistar rats aged 7–9 weeks (weight range: 180–220 g) from our own breeding facilities were used in this study. Rats were housed in regular plastic cages at 22°C–24°C temperature, under a 12 h light-dark cycle and had free access to food and purified water *ad libitum*. During the study, animal suffering was minimized and a minimum number of animals were used. Each rat was used in only one experiment and at the end, the animals were euthanized in a CO_2_ chamber. Experiments were realized according the recommendations on Ethical Standards for Investigation in Animals (Zimmermann, 1983) and the Canadian Council on Animal Care (CCAC) (Olfert et al., 1993). Likewise, the study protocol was revised and accepted by an independent Institutional Animal Care and Use Committee (CINVESTAV, IPN, Ciudad de México, Mexico) with the register number 0169–15.

### 2.2 Drugs

α-Bisabolol, diclofenac, formaldehyde, naloxone (non-selective opioid receptors antagonist), metformin (a drug hypoglycemic biguanide), NG-L-nitro-arginine methyl ester (L-NAME; a NO synthase inhibitor), 1 H-(1,2,4)-oxadiazolo (4,2-a) quinoxalin-1-one (ODQ; a NO-sensitive soluble guanylyl cyclase inhibitor), glibenclamide and glipizide (both ATP-sensitive K^+^ channel blockers; K_ir_6.1-2), 4-aminopyridine (4-AP) and tetraethylammonium chloride (TEA) (both voltage-gated K^+^ channel blockers; K_V_), apamin (a small conductance Ca^2+^-activated K^+^ channel blocker; K_Ca_2.1-2.3), and charybdotoxin (a big conductance Ca^2+^-activated K^+^ channel blocker; K_Ca_1.1), were purchased from Sigma-Aldrich (Toluca, Mexico). *α*-Bisabolol was diluted in 1% Tween 80. Diclofenac, naloxone, metformin, L-NAME, TEA, 4-AP, apamin, and charybdotoxin were dissolved in saline solution. Glibenclamide, glipizide and ODQ were dissolved in a 20% DMSO solution.

### 2.3 Measurement of antinociception

In the present study, nociception and antinociception were determined with the rat paw 1% formalin test (Ortiz et al., 2021; Ortiz et al., 2022). Briefly, 50 μL of a 1% formalin solution were administered subcutaneously (s.c.) in the right hind paw of the rat, and the number of flinches were counted. Nociception was quantified as the number of flinches of the injected paw with formalin during 1-min periods every 5 up to 60 min after injection. The number of flinches yielded a biphasic curve, and the area under the curve was calculated for both phases. To determine the local peripheral antinociception of *α*-Bisabolol, diclofenac and their combination, groups of independent rats at different times were administered with vehicles or increasing doses of *α*-Bisabolol (100, 180,300 and 560 µg/paw at 20 min before), diclofenac (10, 30, 100 and 300 µg/paw at 13 min before), or the *α*-Bisabolol (20 min before)-diclofenac (13 min before) combinations (selected doses with the antinociceptive results of the phase two of individual drugs: 30.7, 61.4, 122.9 and 245.7 µg/paw) s.c. in the right paw before the injection of 50 µL of 1% formalin in the same paw. To demonstrate that the antinociceptions of *α*-Bisabolol, diclofenac and the *α*-Bisabolol-diclofenac combination were locally at the injection site, the highest doses of the drugs were administered in the contralateral (left) paw before 1% formalin was administered to the right paw and the responses were evaluated.

### 2.4 Data analysis

The area under the curve (AUC) of the local peripheral antinociceptive effects produced by each individual and combined drug regimen was calculated as previously described (De Paz-Campos et al., 2014; Ortiz et al., 2016; Ortiz, 2017; Ortiz et al., 2017; Ortiz et al., 2018). The percentage of antinociceptive activity for both phases of the formalin test was obtained according to the following equation (Ortiz et al., 2016; Ortiz, 2017; Ortiz et al., 2017; Ortiz et al., 2018): % antinociception = [(AUC_vehicle_—AUC_post-drug_)/AUC_vehicle_] × 100.

### 2.5 Drug combinations

The antinociceptive effective doses 40 (ED_40_) were calculated as reported by Tallarida (2000). The interaction between *α*-Bisabolol and diclofenac was characterized by isobolographic analysis in which it was assumed that the mixtures are comprised of equieffective dose of the individual drugs (Tallarida, 2000; Ortiz et al., 2016; Ortiz, 2017; Ortiz et al., 2017; Ortiz et al., 2018). In this sense, from the dose–response curves of each individual agent on the phase two of the formalin test (α-Bisabolol or diclofenac), the dose resulting in 40% of the effect (ED_40_) were calculated. After that, a dose–response curve was obtained by concurrent delivery of the two drugs (α-Bisabolol + diclofenac) in a fixed-ratio mixture (1:1) that was based on the ED_40_ values of each individual drug. To produce the experimental antinociceptive effect–dose curve each group of rats received one of the following dose mentioned on Table 1. The antinociceptive experimental ED_40_ value for the *α*-Bisabolol–diclofenac combination was calculated from the phase two of this curve.

**TABLE 1:** Dosing amount of each drug in the combinations according to the ED_40_ of *α*-Bisabolol (311.8 µg/paw) and diclofenac (179.6 µg/paw).

α-Bisabolol		Diclofenac		Total dose
µg/paw		µg/paw		µg/paw
ED_40_/2: 155.9	+	ED_40_/2: 89.8	=	245.7
ED_40_/4: 77.95	+	ED_40_/4: 44.9	=	122.9
ED_40_/8: 39.0	+	ED_40_/8: 22.4	=	61.4
ED_40_/16: 19.5	+	ED_40_/16: 11.2	=	30.7

The theoretical ED_40_ value of the drug mixture was calculated according to the following equation: *α*-Bisabolol + diclofenac combination theoretical ED_40_ = diclofenac ED_40/2_ + *α*-Bisabolol ED_40/2_. This theoretical ED_40_ value was statistically contrasted with the experimentally obtained ED_40_ value (Tallarida, 2000; Ortiz et al., 2016; Ortiz, 2017; Ortiz et al., 2017; Ortiz et al., 2018). To reinforce the analysis of the results, the interaction index (*γ*) value was obtained using the following formula: γ = experimental ED_40_ of combination/theoretical ED_40_ of combination. The γ value indicated the kind of pharmacological interaction as follows: γ = 1, additive interaction; γ > 1, antagonism; γ < 1, synergistic interaction (Tallarida, 2000; Ortiz et al., 2016; Ortiz, 2017; Ortiz et al., 2017; Ortiz et al., 2018).

### 2.6 Design of the study on the mechanism of action of the drug interaction of *α*-Bisabolol and diclofenac

In order to demonstrated the possible participation of a biguanide-dependent mechanism, opioid receptor, the production of NO and cGMP and the activation of K^+^ channels in the *α*-Bisabolol-diclofenac combinations-induced antinociception, groups of independent rats were administered with the *α*-Bisabolol (20 min before formalin) + diclofenac (13 min before formalin) combinations and then the corresponding vehicles or metformin (400 µg/paw), naloxone (50 µg/paw), L-NAME (100 µg/paw), ODQ (100 µg/paw), K^+^ channels blockers 7 min before the administration of formalin, and then the responses were evaluated as previously mentioned. Only the doses of the combinations (α-Bisabolol and diclofenac) that produced statistically significant antinociceptive effects were used to evaluate their probable mechanism of action (α-Bisabolol 155.9 µg/paw + diclofenac 89.8 µg/paw, *α*-Bisabolol 77.95 µg/paw + diclofenac 44.9 µg/paw, and *α*-Bisabolol 39.0 µg/paw + diclofenac 22.4 µg/paw).

All dose of *α*-Bisabolol, diclofenac and blockers were administered in a volume of 50 µL. Each of the rats in all experimental groups received four injections, and the appropriate controls for multiple injections and vehicles were performed. The doses and drug administration schedules of blockers and antinociceptive drugs for peripheral administration were selected based on previous reports (Ortiz, 2012; Ortiz et al., 2021; Ortiz et al., 2022) and on pilot experiments in our laboratory. Rats in all experimental groups were evaluated for possible side reaction.

### 2.7 Statistical analyses

The dose-response curves were evaluated for significance using the one-way Analysis of Variance and Dunnett´s test. The comparison between theoretical and experimental values was realized using the Student’s *t*-test (Tallarida, 2000; Ortiz et al., 2016; Ortiz, 2017; Ortiz et al., 2017; Ortiz et al., 2018). Experimental ED_40_ values significantly inferior to the theoretical ED_40_ values, respectively, indicated a synergistic interaction. The results were considered statistically significant when *p* < 0.05.

## 3 Results

### 3.1 Antinociception induced by *α*-Bisabolol and diclofenac

**FIGURE 1:**
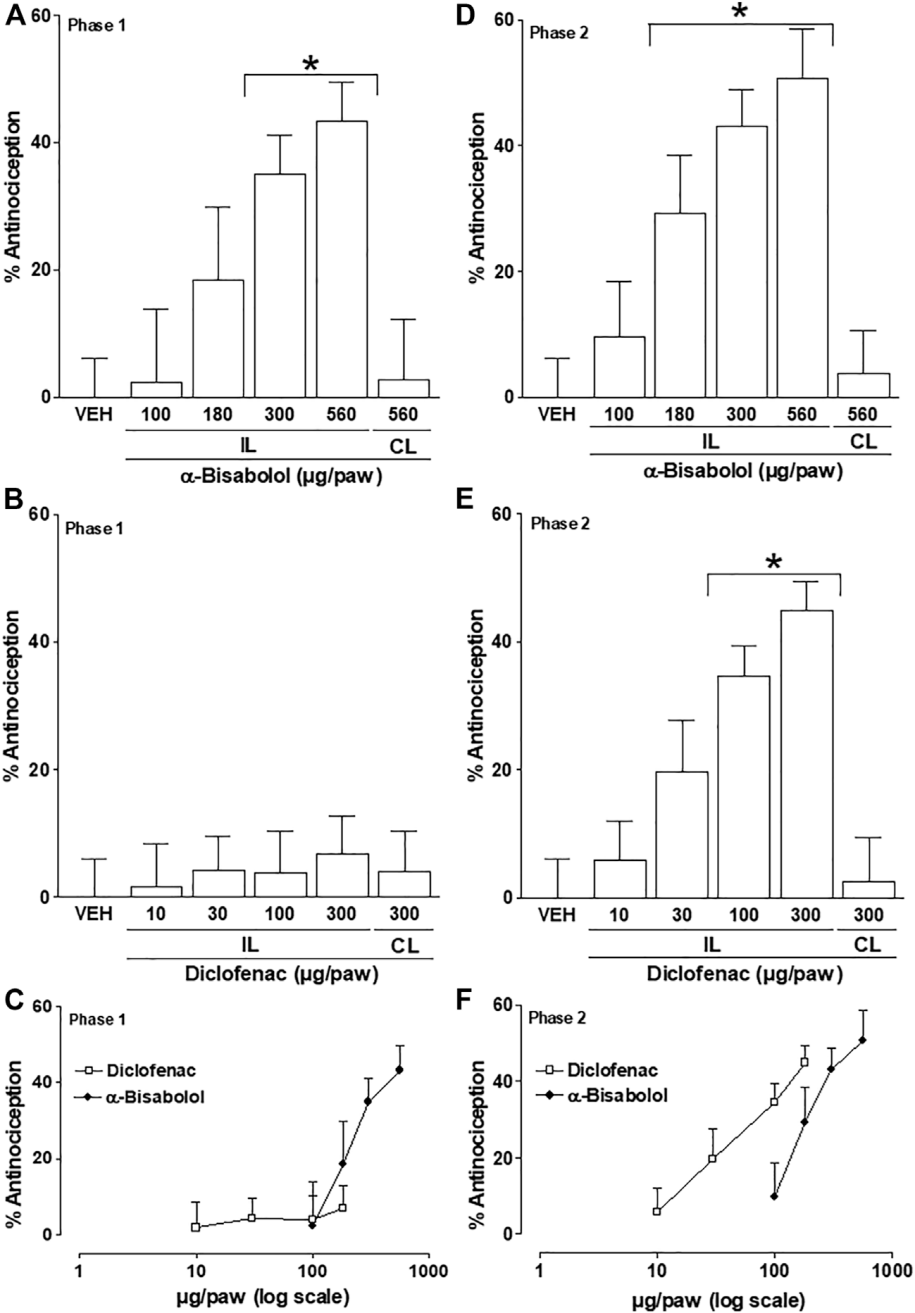
Local peripheral antinociception of *α*-Bisabolol and diclofenac on both phases of the 1% rat formalin test. Earlier to the injection of formalin, rats were pretreated with a local peripheral injection of vehicle (VEH), *α*-Bisabolol or diclofenac. Results are expressed as the antinociception percent on phase one **(A–C)** and phase two **(D–F)**. Results are presented in bar graphics in **(A, B, D, E)**. Data from the previous results are presented in sigmoid curves in **(C, F)** (log scales). Each point corresponds to the mean ± SEM of 5-6 animals. *Significant difference from vehicle group (*p* < 0.05) as determined by the analysis of variance followed by Dunnett’s test.

The local peripheral administration of 1% formalin produced a flinching conduct indicative of nociceptive effect. Ipsilateral, but not contralateral, local peripheral injection of *α*-Bisabolol, but not diclofenac, decreased significantly the phase one of the formalin test (Compared with the effects produced by the respective vehicles. P < 0.05; Figures 1A–C). The maximum effect (E_max_) achieved with *α*-Bisabolol on the phase one was 43.3%. On the other hand, ipsilateral, but not contralateral, local administration of *α*-Bisabolol or diclofenac produced dose-dependent antinociception during the second phase of the test (Compared with the effects produced by the respective vehicles. P < 0.05; Figures 1D–F). The maximum effects (E_max_) achieved with *α*-Bisabolol and diclofenac on the phase two were 50.7% and 44.9%, respectively. The ED_40_ values of individual administrations of *α*-Bisabolol and diclofenac on the phase two were 311.8 ± 32.8 µg/paw and 179.6 ± 14.1 µg/paw, respectively.

### 3.2 Interactions between *α*-Bisabolol and diclofenac

**FIGURE 2:**
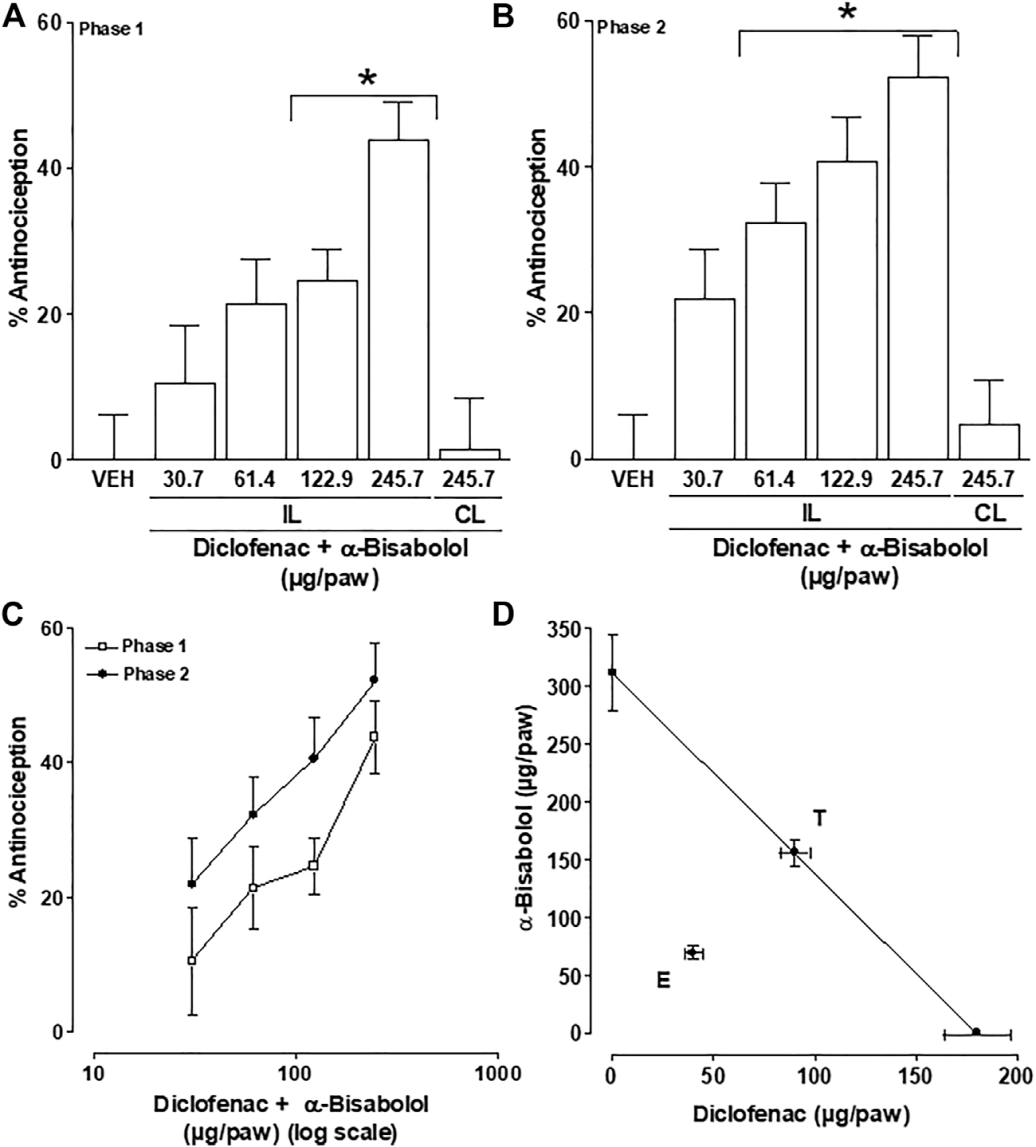
Drug interaction study: Local peripheral antinociception of the *α*-Bisabolol-diclofenac combination on the phase one **(A)** and phase two **(B)** of the formalin test; rats were pretreated with the local injections of vehicle (VEH) or *α*-Bisabolol-diclofenac combinations before formalin injection. Data from **(A, B)** are presented in sigmoid curves in **(C)** (log scales). **(D)** Isobologram showing the local interaction between *α*-Bisabolol and diclofenac on the second phase of the formalin test. The oblique line between the *x* and *y*-axes is the theoretical additive line. The point in the middle of this line, indicated by “T”, is the theoretical additive point calculated from the individual drug ED values. The experimental point indicated by “E”, is the actual observed ED value for this combination. Horizontal and vertical bars indicate SEM.

In independent groups of rats, ipsilateral, but not contralateral, local peripheral injection of fixed-dose ratio (1:1) combinations of *α*-Bisabolol and diclofenac (at the doses mentioned in Table 1) produced dose-dependent antinociception during both phases of the formalin test (Compared with the effects produced by the respective vehicles. *p* < 0.05). These antinociceptive effects were used to construct dose-response curves (Figure 2A=Phase one, 2B = Phase two and 2C = Both phases). The maximum effects (E_max_) achieved with *α*-Bisabolol-diclofenac combinations on the phase one and the phase two were 43.8% and 52.2%, respectively.

The final experimental ED_40_ value from the phase two of the formalin test (Figures 2B, C) was 109.2 ± 8.5 µg/paw, for local peripheral antinociception. This experimental ED_40_ was significantly lower (*p* < 0.05) than the theoretical ED_40_ value of 245.7 ± 17.8 µg/paw, for the local peripheral antinociception. The presence of synergistic interaction is shown in Figure 2D, by the experimental ED_40_ (E) value being below the additive effect threshold (T). Furthermore, the interaction index (*γ*) value for the *α*-Bisabolol-diclofenac combination (0.44 ± 0.04) was significantly different from unity (*p* < 0.05). This result suggests that at local peripheral level, the interaction between *α*-Bisabolol and diclofenac is synergistic in terms of antinociceptive action.

### 3.3 Effect of the naloxone, metformin and NO-cGMP-K^+^ channel pathway inhibitors on the *α*-Bisabolol-diclofenac combination-induced antinociception

**FIGURE 3:**
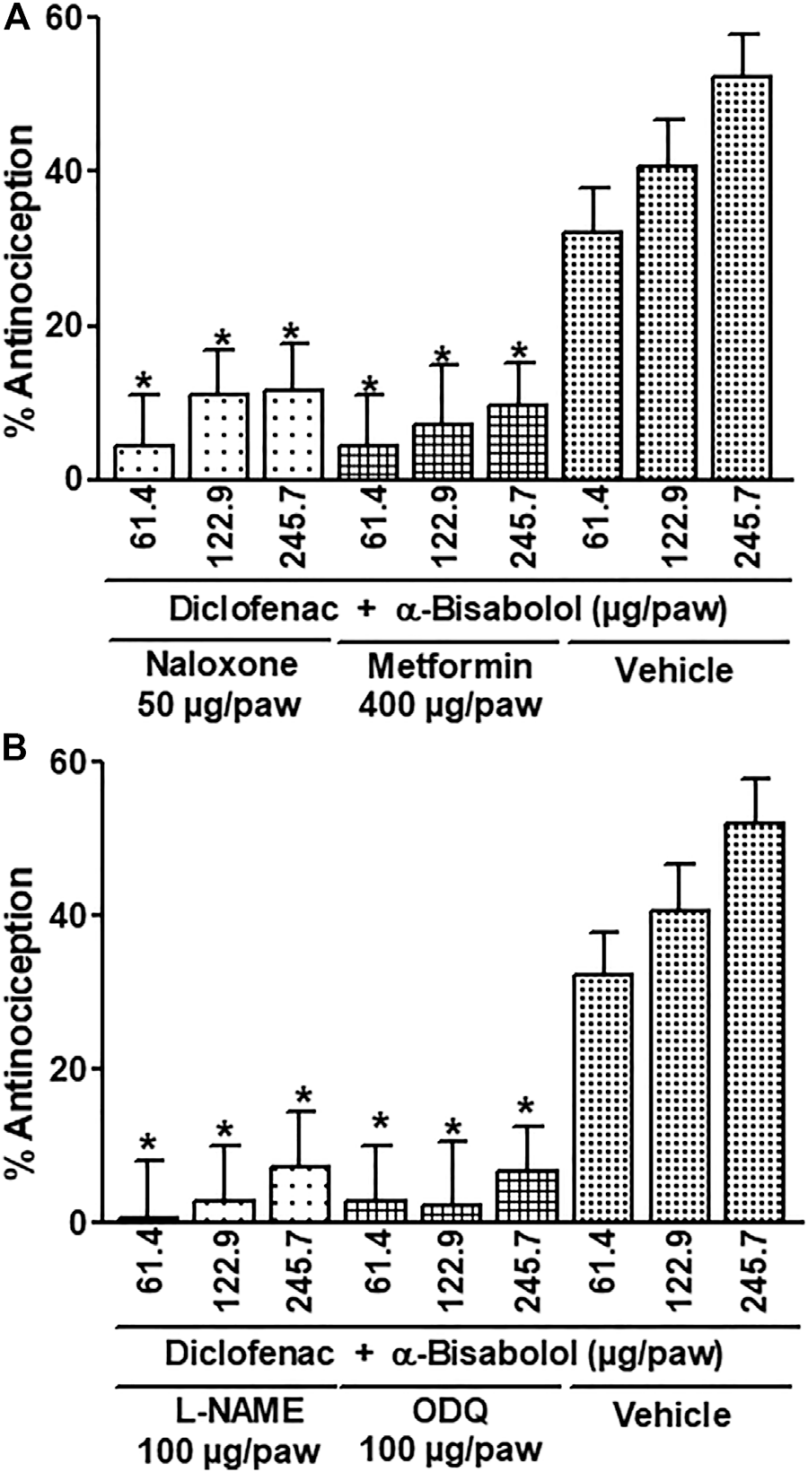
Effect of the local peripheral injection of naloxone **(A)**, metformin **(A)**, L-NAME **(B)** or ODQ **(B)** on the antinociceptive activity of the *α*-Bisabolol-diclofenac combinations during the formalin test. Rats received a local injection of *α*-Bisabolol (−20 min), diclofenac (−13 min) and then the inhibitors (−7 min) into the right paw. Results are expressed as the antinociception percent on phase two. Each point corresponds to the mean ± SEM of 5-6 animals. *Significant difference from vehicle group (*p* < 0.05) as determined by the analysis of variance followed by Dunnett’s test.

The local peripheral administration of naloxone (50 µg/paw) and metformin (400 µg/paw) significantly reduced between 70 and 85 percent of the antinociceptive effects produced by the diclofenac + *α*-Bisabolol combinations during the phase two of the formalin test (Compared with the effects produced by the respective vehicles (*p* < 0.05; Figure 3A). Likewise, the local injection of L-NAME and ODQ decreased by 85%–95% the antinociception produced by the combinations evaluated on the second phase of the formalin test (Contrasted with the results produced by the correspondent vehicles (*p* < 0.05; Figure 3B).

**FIGURE 4:**
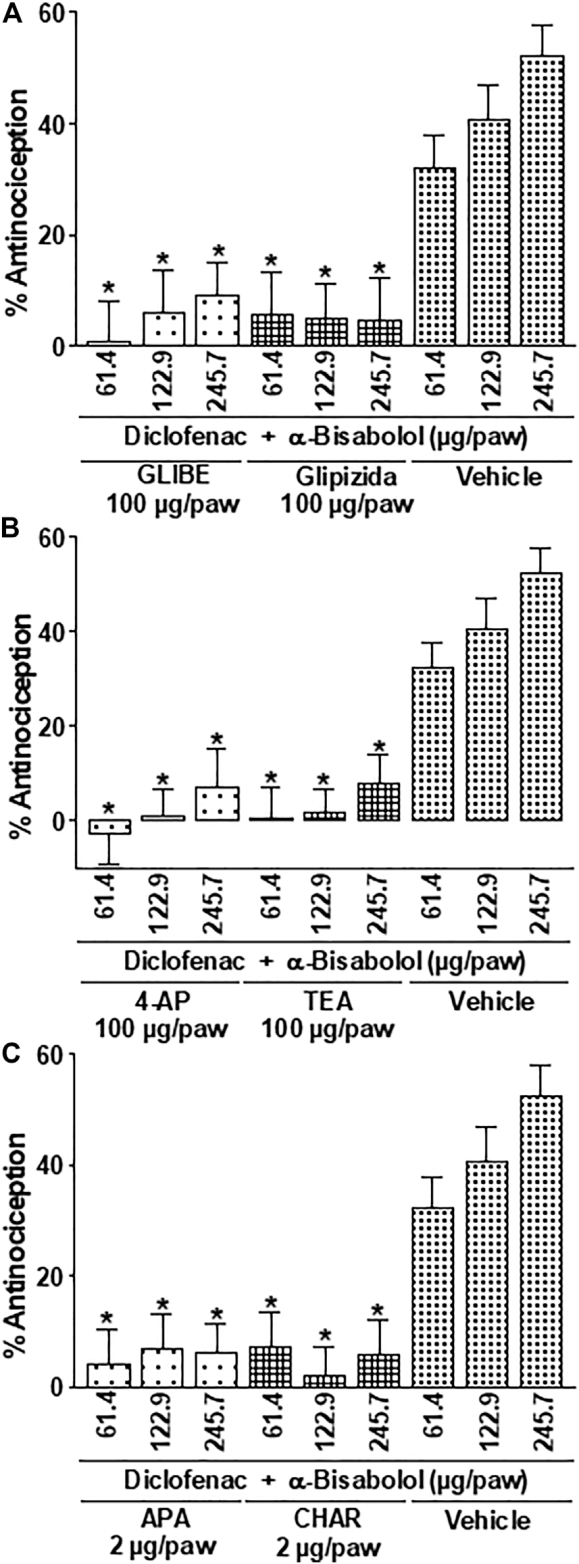
Effect of the local peripheral injection of glibenclamide (GLIBE) (A), glipizide **(A)**, 4-AP **(B)**, TEA **(B)**, apamin **(C)** or charybdotoxin (CHAR) **(C)** on the antinociceptive activity of the *α*-Bisabolol-diclofenac combinations during the formalin test. Rats received a local injection of *α*-Bisabolol (−20 min), diclofenac (−13 min) and then the inhibitors (−7 min) into the right paw. Results are expressed as the antinociception percent on phase two. Each point corresponds to the mean ± SEM of 5-6 animals. *Significant difference from vehicle group (*p* < 0.05) as determined by the analysis of variance followed by Dunnett’s test.

Similarly, the local peripheral injection of the ATP-sensitive K^+^ channel blockers (K_ir_6.1-2) glibenclamide and glipizide, the voltage-gated K^+^ channel blockers (K_V_) 4-AP, TEA, and the Ca^2+^-activated K^+^ channel blockers apamin or charybdotoxin, significantly decreased the mixtures induced-antinociceptive effects by 70%–100% during the phase two of the formalin test (Compared with the effects produced by the respective vehicles (*p* < 0.05; Figures 4A–C).

## 4 Discussion

Skin can work as a drugs reservoir and a place to their metabolism. The skin is an immunologically active organ and drugs administered to the skin surface can be absorbed at different depths and produce local, subcutaneous or adjacent deep site pharmacological effects or even systemic effects (Ruela et al., 2016). The topical or local anti-inflammatory and analgesic effects in the treatment of painful and inflammatory conditions are currently of great interest due to their few adverse effects compared to systemic therapy (Goh and Lane, 2014; Iwaszkiewicz and Hua, 2014; Ruela et al., 2016; Ansari et al., 2019). This strategy is reinforced and improved if low doses of the drugs are administered and, moreover, they produce few undesired effects. In this sense, the combined administration of two or more drugs is performed to achieve synergism or potentiation of the beneficial pharmacological effect. The aspirin-paracetamol-caffeine combination for the management of migraine or the amitriptyline-ketamine topical combination for treatment of rectal, perineal and genital pain are clinical examples of drug-combinations (Poterucha et al., 2012; Diener et al., 2022). In the present study, the isobolographic examination found a significant synergism of the local peripheral administration of the *α*-Bisabolol-diclofenac combination. This local peripheral synergism observed agree with the systemic synergistic interaction of a *Matricaria chamomilla* ethanolic extract (MCE) or *α*-Bisabolol with diclofenac on nociception, inflammation and gastric damage in rats (Ortiz et al., 2016; Ortiz et al., 2018). Likewise, other studies have demonstrated an antinociceptive synergistic interaction of diclofenac with other plant constituents, such as geraniin or xylopic acid (Woode et al., 2015; Boakye-Gyasi et al., 2018). Therefore, the combination of diclofenac with *α*-Bisabolol could be a good option for the management of pain in inflammatory processes. However, the combination of *α*-Bisabolol and diclofenac should be evaluated in clinical situations in order to ensure its safety and efficacy in the management of acute and inflammatory pain.

Individual local peripheral action mechanisms of *α*-Bisabolol and diclofenac involve the activation of the NO-cGMP-K^+^ channel pathway (Ortiz et al., 2003; Ortiz et al., 2021; Ortiz et al., 2023). On the other hand, opioid receptors are involved in the local peripheral antinociception produced diclofenac, but not of *α*-Bisabolol (Silva et al., 2016; Melo et al., 2017; Ortiz et al., 2021). Therefore, the second main objective in the present study, was to determine the activation of the opioid receptor-NO-cGMP-K^+^ channel pathway in the synergistic antinociception of the *α*-Bisabolol-diclofenac combination in the formalin test.

Opioid agonists produce their effects through the activation of various types of receptors: µ, mu or MOP; δ, delta or DOP; κ, kappa or KOP; or opioid receptor-like or NOP (Stein, 2016). All opioid receptors are coupled to the G_i_ proteins and their activation decrease cyclic adenosine monophosphate production, increase K^+^ conduction and blockade of Ca^2+^ currents. These actions cause both presynaptic inhibition of neurotransmitter release from the central endings of small-diameter primary afferent fibers and postsynaptic inhibition of membrane depolarization of nociceptive dorsal horn neurons (Stein, 2016). Previous studies demonstrated the participation of opioid receptors in the local peripheral antinociception produced by several non-opioid drugs, such as diclofenac, dipyrone, docosahexaenoic acid, or pamabrom (Silva et al., 2016; Landa-Juárez et al., 2019; Ortiz et al., 2022). However, the activation probable of opioid receptors in the *α*-Bisabolol induced-antinociception was disqualified (Melo et al., 2017; Ortiz et al., 2021). In the present study, the *α*-Bisabolol-diclofenac combination-induced antinociception was reverted significantly by the opioid receptor antagonist naloxone. It is probable that the participation of opioid receptors in this special situation is mainly produced by diclofenac. In the present study, a dose–response curve by concurrent delivery of the two drugs (α-Bisabolol + diclofenac) in a fixed-ratio mixture (1:1) was performed. Therefore, experiments with different fixed-ratio *α*-Bisabolol-diclofenac combinations (1:3, 1:10, 3:1 or 10:1) are necessary to accurately determine the participation of opioid receptors in the present antinociceptive combination. Local peripheral administration of L-NAME, ODQ and potassium channel blockers were able to decrease the antinociceptive effect induce by the *α*-Bisabolol-diclofenac combination. This result confirm the activation of the opioid receptor-NO-cGMP-K^+^ channel pathway.

Our results suggest the presence, in subcutaneous tissue nociceptors, of ATP-sensitive-, Ca^2+^-activated- and voltage-gated K^+^ channels that are activated or controlled by cGMP. Due to the K^+^ gradient (produced by the Na^+^/K^+^-ATPase), the opening of the K^+^ channel results in an abrupt outward release of K^+^, causing hyperpolarization (Higerd-Rusli et al., 2023). In this sense, hyperpolarization desensitizes the neuron and prevents it from being activated or depolarized by another stimulus during this time, or at least raises the threshold for any new stimulus (Higerd-Rusli et al., 2023). Thus, hyperpolarization prevents depolarizations from occurring, thus avoiding the transmission and conduction of messages to second or third order neurons. In the end, the hyperpolarization caused by the opening of K^+^ channels produces a decrease in nociception to the applied stimulus.

Metformin, a biguanide, is a first-line hypoglycemic agent in the treatment of type 2 diabetes. Its pharmacological effect includes inhibition of hepatic gluconeogenesis, by reducing the expression of various genes involved. Likewise, metformin inhibits mitochondrial glycerol-3-phosphate dehydrogenase (Cao et al., 2022). In the present study, the synergistic antinociception of the *α*-Bisabolol-diclofenac combination was significantly blocked by metformin. This result agrees with the activation of a biguanide-dependent mechanism in the antinociception induced by diclofenac (Ortiz, 2012).

There are some limitations in the present study. Firstly, the antinociceptive curve of the combination *α*-Bisabolol + diclofenac was obtained only with a fixed ratio mixture (1:1). This pharmacological strategy has been widely used to demonstrate the drug interaction of various drugs (Tallarida, 2000; Poterucha et al., 2012; De Paz-Campos et al., 2014; Ortiz et al., 2016; Ortiz, 2017; Ortiz et al., 2017; Aweke et al., 2018; Ortiz et al., 2018; Shep et al., 2020; Diener et al., 2022). Therefore, the pharmacological synergy observed in the present study is important as a first evidence of their peripheral antinociceptive effects when combined. However, it is recommended to evaluate experimentally different fixed ratios of *α*-Bisabolol-diclofenac combinations (1:3, 1:10, 3:1 or 10:1) to determine the type of drug interactions and the likely involvement of other mechanisms of action. Another limitation is the lack of evaluation of the GI and VC injuries with the *α*-Bisabolol-diclofenac combinations. Experimental designs on gastric damage have shown the presence of GI lesions 3 h after systemic administration of diclofenac (Ortiz et al., 2017; Ortiz et al., 2018). However, we did not find any GI lesions of the peripheral local diclofenac within 3 h of the experiments in the present study (data not shown). Likewise, a previous study demonstrated the lack of GI damage from systemic administration of *α*-Bisabolol at 3 and 6 h in rats (Ortiz et al., 2018). For these reasons, in the present study we did not evaluate the GI damage by local peripheral administration of *α*-Bisabolol or the combinations. On the other hand, the CV injury by non-steroidal anti-inflammatory drugs is produced with their chronic or prolonged administration (Kearney et al., 2006). In the present study, because only a single low dose of diclofenac was administered to each rat, it was not necessary to assess CV damage.

## 5 Conclusion

The present results demonstrated a significant peripheral antinociceptive potentiation effect of the *α*-Bisabolol-diclofenac combination in the formalin test. This synergism was due to activation of the opioid receptor-NO-cGMP-K^+^ channel pathway. Likewise, the *α*-Bisabolol-diclofenac combination could activate a biguanide-dependent mechanism to generate antinociception at the peripheral level. Further research into the efficacy and gastric security resulting from the administration of this combination in different experimental models and clinical situations is awaiting confirmation.

## Data Availability

The raw data supporting the conclusion of this article will be made available by the author, without undue reservation.

## Abbreviations

Nitric oxide, NO; cyclic guanylyl monophosphate, cGMP; gastrointestinal, GI; cardiovascular, CV; NG-nitro-L-arginine methyl ester, L-NAME; 1H- (1,2,4)-oxadiazolo (4,2-a) quinoxalin-1-one, ODQ; 4-aminopyridine, 4-AP; tetraethylammonium chloride, TEA; area under the curve, AUC; effective dose, ED.
